# Sulfide level in municipal sludge digesters affects microbial community response to long-chain fatty acid loads

**DOI:** 10.1186/s13068-019-1598-1

**Published:** 2019-11-02

**Authors:** Sepehr Shakeri Yekta, Tong Liu, Mette Axelsson Bjerg, Luka Šafarič, Anna Karlsson, Annika Björn, Anna Schnürer

**Affiliations:** 1 Department of Thematic Studies-Environmental Change and Biogas Research Center, Linköping University, 581 83 Linköping, Sweden; 2 Department of Molecular Sciences, Uppsala BioCenter, Swedish University of Agricultural Sciences, 75007 Uppsala, Sweden; 3 Scandinavian Biogas Fuels AB, Stockholm, Sweden

**Keywords:** Anaerobic digestion, Primary and activated sewage sludge, Oleate, Stearate, Sulfide, 16S rRNA gene amplicon sequencing

## Abstract

**Background:**

Waste lipids are attractive substrates for co-digestion with primary and activated sewage sludge (PASS) to improve biogas production at wastewater treatment plants. However, slow conversion rates of long-chain fatty acids (LCFA), produced during anaerobic digestion (AD), limit the applicability of waste lipids as co-substrates for PASS. Previous observations indicate that the sulfide level in PASS digesters affects the capacity of microbial communities to convert LCFA to biogas. This study assessed the microbial community response to LCFA loads in relation to sulfide level during AD of PASS by investigating process performance and microbial community dynamics upon addition of oleate (C_18:1_) and stearate (C_18:0_) to PASS digesters at ambient and elevated sulfide levels.

**Results:**

Conversion of LCFA to biogas was limited (30% of theoretical biogas potential) during continuous co-digestion with PASS, which resulted in further LCFA accumulation. However, the accumulated LCFA were converted to biogas (up to 66% of theoretical biogas potential) during subsequent batch-mode digestion, performed without additional substrate load. Elevated sulfide level stimulated oleate (but not stearate) conversion to acetate, but oleate and sulfide imposed a synergistic limiting effect on acetoclastic methanogenesis and biogas formation. Next-generation sequencing of 16S rRNA gene amplicons of bacteria and archaea showed that differences in sulfide level and LCFA type resulted in microbial community alterations with distinctly different patterns. Taxonomic profiling of the sequencing data revealed that the phylum Cloacimonetes is likely a key group during LCFA degradation in PASS digesters, where different members take part in degradation of saturated and unsaturated LCFA; genus W5 (family Cloacimonadaceae) and family W27 (order Cloacimonadales), respectively. In addition, LCFA-degrading *Syntrophomonas*, which is commonly present in lipid-fed digesters, increased in relative abundance after addition of oleate at elevated sulfide level, but not without sulfide or after stearate addition. Stearate conversion to biogas was instead associated with increasing abundance of hydrogen-producing *Smithella* and hydrogenotrophic *Methanobacterium*.

**Conclusions:**

Long-chain fatty acid chain saturation and sulfide level are selective drivers for establishment of LCFA-degrading microbial communities in municipal sludge digesters.

## Background

Biogas production via anaerobic digestion (AD) of sewage sludge contributes to the supply of locally produced renewable energy, reducing the demand for fossil fuels [1]. In Sweden, anaerobic digester units at wastewater treatment plants (WWTP) account for approximately half of the total number of biogas plants, providing one-third of all biogas produced [2]. Increasing biogas production at WWTP is of strategic importance, considering Sweden’s goal of achieving zero greenhouse gas emissions by 2045 [3]. The excess capacity of existing anaerobic digesters at WWTP can be used to increase biogas production by co-digestion of energy-rich substrates, among which waste lipids are attractive co-substrates due to their high methane potential [4]. However, introduction of lipids into anaerobic digesters may perturb process performance due to accumulation of long-chain fatty acids (LCFA) formed as intermediate degradation products of lipids [5].

Anaerobic degradation of LCFA is slow because of mass transfer limitations imposed by the hydrophobic nature of LCFA and the slow growth of LCFA-degrading microorganisms [5]. As a result, continuous supplementation of lipid-rich substrates to anaerobic digesters may cause LCFA accumulation, which in turn can lead to operational disturbances such as sludge foaming, inhibition of microbial activities, acidification, and ultimately process failure [5]. To tackle the slow degradation kinetics of lipids, development of special reactor designs and operating conditions, such as two-phase anaerobic digesters, inverted anaerobic sludge reactors, and alteration of feeding frequency, has been suggested [6–8]. However, limited knowledge of in situ parameters regulating anaerobic LCFA degradation and associated process disturbances discourages the use of waste lipids for improving biogas production at existing biogas plants at WWTP.

Long-chain fatty acids, such as oleate, stearate, and palmitate are commonly present in lipid-fed anaerobic digesters, where their conversion to biogas is carried out by proton-reducing acetogenic bacteria via the cyclic β-oxidation pathway [9]. During β-oxidation of LCFA, two carbon atoms are cleaved from the LCFA structure at each cycle, resulting in production of mainly acetate and hydrogen/formate. The thermodynamic feasibility of the β-oxidation pathway relies on low partial pressure of hydrogen, necessitating syntrophic association of LCFA-degrading bacteria with hydrogen-utilizing microorganisms. Among the microbial groups commonly established in anaerobic digesters, syntrophic bacteria belonging to the Syntrophomonadaceae and Syntrophaceae seem to play a central role in conversion of LCFA to biogas [9, 10]. Both saturated and unsaturated LCFA are degraded via the syntrophic β-oxidation pathway, whereas a limited number of β-oxidizing bacteria have been associated with degradation of unsaturated LCFA (e.g., *Syntrophomonas* and *Thermosyntrophica* within the family Syntrophomonadaceae), most likely because of the need for carbon chain saturation as part of the degradation process [11].

In a previous study, we observed that an increase in sulfur (S) level during AD of sewage sludge by direct supplementation of sulfide favored growth of the LCFA-degrading *Syntrophomonas* genus, allowing for 25% faster conversion of oleate to biogas compared with the control [12]. To our knowledge, the direct effects of sulfide on β-oxidation of LCFA in anaerobic digesters have not yet been identified. However, the importance of sulfide formation via sulfate reduction has been pointed out as a regulatory parameter for LCFA conversion to biogas. An inhibitory effect of biogenic sulfide formation on conversion of LCFA to biogas has been observed, and attributed to growth of sulfate-reducers in syntrophic interaction with β-oxidizing microorganisms, which outcompete the methanogens [11]. Contrasting results have also been reported, e.g., that co-digestion of sulfate-rich substrate with lipids and formation of sulfide has only minor effects on methanogenesis and biogas formation [13]. To further elucidate whether the presence of sulfide affects conversion of LCFA to biogas in municipal sludge digesters, in the present study, we assessed the microbial community response to saturated and unsaturated LCFA loads at elevated sulfide level during AD of primary and activated sewage sludge (PASS).

## Methods

### Experimental setup

Six continuous stirred-tank anaerobic digesters, designated F1–F6, each with a working volume of 6 l were inoculated with sludge from a full-scale anaerobic digester at Henriksdal WWTP in Stockholm, Sweden. The digesters were fed with PASS from the same WWTP, collected on one occasion and stored in 10-l containers at − 20 °C. The laboratory-scale digesters were operated at mesophilic conditions (37 °C) with a PASS loading rate of 1.2–1.5 g volatile solids (VS) l^−1^ d^−1^ (80% primary sludge and 20% activated sludge on a volume basis) and a hydraulic retention time of 20 days, corresponding to the operating conditions of the original digester at Henriksdal WWTP. The variation in the PASS loading rate was due to differences in total solids (TS) and VS content of the substrate aliquots used for feeding during different operating periods.

**Fig. 1:**
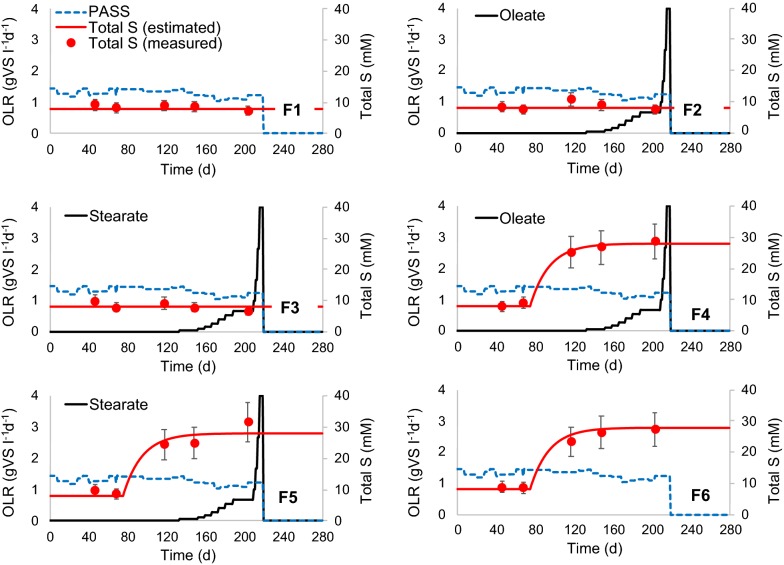
Experimental setup showing the organic loading rate (OLR) of primary and activated sewage sludge (PASS), oleate, and stearate, together with the estimated and measured concentrations of total sulfur (S), in laboratory-scale digesters F1, F2, F3, F4, F5, and F6

From day 76, sodium sulfide (Na_2_S·9H_2_O in de-aerated milliQ water), corresponding to 20 mM S in the substrate, was added daily to digesters F4, F5, and F6. The amount of sulfide added was based on the iron (Fe) content of PASS. Interactions between sulfide and Fe in anaerobic digesters can be indicated by the S:Fe molar ratio [14]. An S:Fe ratio < 1 represents excess of Fe over sulfide in the digester, which has been shown to mitigate potential process disturbances related to occurrence of sulfide by formation of poorly soluble iron sulfide minerals in the solid phase [12]. In the present study, sulfide was added to achieve a S:Fe molar ratio of 0.9 in F4, F5, and F6. Total S content of the sulfide-amended digesters increased from 8 to a final level of 28 mM, while total S in F1, F2, and F3 remained constant at 8 mM (Fig. 1), equivalent to an S:Fe molar ratio of 0.3. Between days 133 and 208, an unsaturated LCFA, oleate (C_18:1_), was added to F2 and F4 at levels corresponding to a stepwise increase in the loading rate from 0.0 to 0.67 g VS l^−1^ d^−1^, followed by an increase to 4.0 g VS l^−1^ d^−1^ between days 209 and 218 (Fig. 1). A saturated LCFA, stearate (C_18:0_), was added to F3 and F5 at the same loading rates. Digesters F1 and F6 were operated as controls and received PASS as the only substrate. The PASS and LCFA loadings were stopped on day 219, to evaluate the potential for conversion of the undegraded LCFA to biogas during batch-mode digestion (Fig. 1).

### Process monitoring

Laboratory-scale digesters (Belach Bioteknik, Skogås, Stockholm, Sweden) were equipped with gas meters, working on the principle of liquid displacement, for monitoring biogas production. Volumetric biogas production is reported at standard conditions (0 °C and 1.013 bar). Composition of the biogas (i.e., methane, carbon dioxide, oxygen, and hydrogen sulfide) was determined weekly during continuous feeding of the substrate (days 0–218), using a portable gas analyzer (Biogas Check, Geotech, Chelmsford, UK). Twice a week, the pH was measured by a pH meter (InoLab 7310, WTW, Weilheim, Germany) and concentrations of volatile fatty acids (VFA; acetate, propionate, butyrate, isobutyrate, valerate, isovalerate, caproate, and isocaproate) were quantified using a gas chromatograph (6890 Series, Hewlett Packard, USA) according to Jonsson and Boren [15]. The TS and VS content of the digester sludge were regularly measured according to the Swedish Standard method SS028113. Total S and Fe concentrations were determined on five occasions (days 46, 68, 117, 148, and 203) by Eurofins Environment Testing Sweden AB (Lidköping, Sweden) according to the Swedish Standard method SS028150-2.

### Microbial community analysis

Triplicate samples were retrieved for microbial community analysis on days 0 (inoculum), 72 (before addition of sulfide), 100, 128 (before addition of LCFA), 168, 177, 205 (during stepwise increase and before overload of LCFA), 218 (after overload of LCFA), 225, 233, 254, 261, and 278 (during batch digestion). DNA was extracted from the samples using the FastDNA spin kit for soil (MP Biomedicals, Santa Ana, CA, USA) and quantified by a Qubit 4 Fluorometer (Invitrogen, Thermo Fisher Scientific, Waltham, MA, USA). Polymerase chain reaction (PCR) was used for amplification of the 16S rRNA genes, using primer pair 515′F(GTGBCAGCMGCCGCGGTAA)/805R(GACTACHVGGGTATCTAATCC) for generation of the bacteria sequence library and 516F(TGYCAGCCGCCGCGGTAAHACCVGC)/915R(GTGCTCCCCCGCCAATTCCT) for generation of the archaea sequence library [16, 17]. Reader is referred to Müller et al. [18] and Shakeri Yekta et al. [12] for details of the PCR procedure used for amplification of the 16S rRNA genes.

Samples were further processed for next-generation amplicon sequencing by Illumina MiSeq technology at the SNP&SEQ Technology Platform of the SciLifeLab in Uppsala, Sweden. Taxonomic profiles of the samples were assigned based on amplicon sequence variants (ASV) after processing the raw sequencing data by DADA2 software [19, 20] and the rRNA database SILVA, release 132 [21]. The raw sequencing data were submitted to the National Center for Biotechnology Information database, with identification number SRP188169. In further analyses, archaea sequences were removed from the bacterial sequence library and bacteria sequences were removed from the archaea sequence library. Bacteria and archaea diversities were evaluated by calculation of first-order Hill’s diversity (^1^D) and evenness (^1^E), based on the reported relevance of these indices for microbial diversity representation [22]. The phylogenetic distances of the microbial communities among the samples were evaluated by weighted UniFrac principal coordinate analysis (PCoA) [23]. Indicator species analyses were also carried out separately on the bacteria and archaea datasets, to statistically assess the occurrence of taxonomic groups in the samples based on relative ASV read counts and frequency of occurrence in individual clusters [24]. The indicator species analysis was performed after K-means clustering of Hellinger-transformed ASV reads and optimization of the number of clusters according to Calinski–Harabasz criterion clustering [25, 26]. Statistical analyses were performed by R software [27], using the vegan package [28].

## Results

### Conversion of LCFA to biogas during co-digestion with PASS

**Fig. 2:**
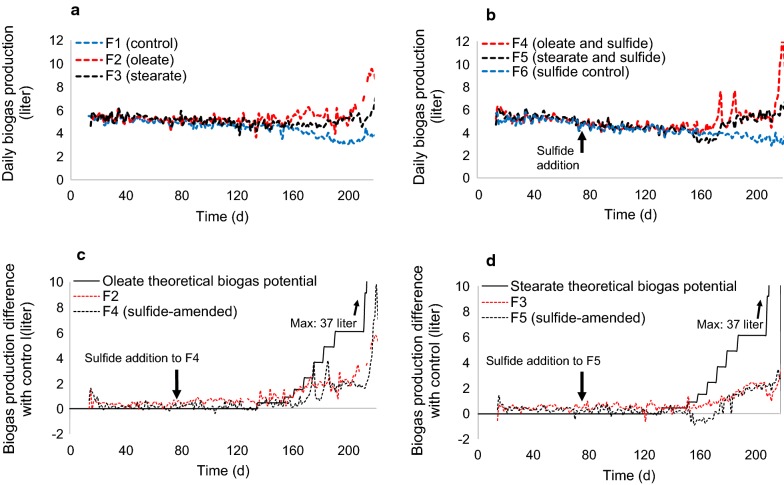
Daily biogas production in digesters F1, F2, and F3 (**a**) and sulfide-amended digesters F4, F5, and F6 (**b**) during continuous feeding of primary and activated sewage sludge. **c** Theoretical biogas potential of oleate, and differences in daily biogas production between oleate-amended digesters (F2 and F4) and control digesters (F1 and F6, respectively). Oleate was added to F2 and F4 from day 133 onward. **d** Theoretical biogas potential of stearate, and differences in daily biogas production between stearate-amended digesters (F3 and F5) and control digesters (F1 and F6, respectively). Stearate was added to F3 and F5 from day 133 onward. Theoretical biogas production values were calculated based on the daily amount of long-chain fatty acids added, using the Buswell equation [51]. The arrow marks the time when sulfide was introduced to F4, F5, and F6 (day 76)

During the start-up phase of the experiment (day 0–75), average daily biogas production from PASS ranged between 5.0 ± 0.3 and 5.4 ± 0.4 l d^−1^ for the six digesters (Fig. 2a, b), corresponding to average daily biogas yields of 620 ± 55 to 680 ± 60 ml g^−1^VS_input_ with a methane content of 60–63% (Table 1). Mean specific biogas production was generally lower between day 73 and 132 than in the start-up phase for all digesters. Thus, there were no apparent differences in biogas production that could be attributed to addition of sulfide to F4, F5, and F6 in this period (Table 1). Following addition of oleate to F2 and stearate to F3, daily biogas production gradually increased by approximately 2.0 l d^−1^ compared with the control when the LCFA loading rate reached 0.67 g VS l^−1^ d^−1^ on day 208 (Fig. 2c, d). However, biogas production was initially higher in F2 than in F3 upon stepwise increase in the LCFA loading rate, indicating faster conversion of oleate than stearate to biogas (Fig. 2c, d).

**Table 1:** Operating parameters monitored during continuous feeding of primary and activated sewage sludge to digesters F1–F6

	Days^a^	F1	F2	F3	F4	F5	F6
Specific biogas production (ml g^−1^VS_input_)	0–75	620 ± 55	645 ± 65	650 ± 60	645 ± 65	655 ± 65	680 ± 60
	76–132	540 ± 20	580 ± 30	585 ± 40	525 ± 35	530 ± 35	550 ± 20
	133–218	525 ± 65	570 ± 100	500 ± 100	505 ± 60	450 ± 90	575 ± 60
Methane (% of biogas)	0–75	61 ± 0	61 ± 0	61 ± 0	61 ± 1	63 ± 1	63 ± 0
	76–132	63 ± 1	62 ± 2	62 ± 3	64 ± 1	62 ± 4	64 ± 1
	133–218	61 ± 1	62 ± 1	61 ± 1	63 ± 1	62 ± 1	63 ± 1
Gaseous H_2_S (ppm)	0–75	< 20	< 20	< 20	< 20	< 20	< 20
	76–132	< 20	< 20	< 20	280 ± 100	310 ± 115	250 ± 105
	133–218	< 20	< 20	< 20	510 ± 100	440 ± 70	470 ± 60
Total solids (% of total weight)	0–75	2.1 ± 0.2	1.9 ± 0.2	2.1 ± 0.2	2.1 ± 0.2	2.1 ± 0.2	2.1 ± 0.2
	76–132	2.1 ± 0.1	2.2 ± 0.1	2.1 ± 0.1	2.3 ± 0.1	2.3 ± 0.2	2.3 ± 0.2
	133–218	2.0 ± 0.2	2.1 ± 0.2	2.2 ± 0.3	2.2 ± 0.3	2.4 ± 0.2	2.2 ± 0.0
Volatile solids (% of TS)	0–75	62 ± 1	62 ± 1	63 ± 1	63 ± 1	62 ± 2	63 ± 1
	76–132	61 ± 1	61 ± 1	62 ± 2	58 ± 2	57 ± 4	58 ± 2
	133–218	62 ± 0	63 ± 1	65 ± 3	57 ± 1	59 ± 3	56 ± 0

^a^Days 0-75: start-up phase; Day 76: start of sulfide addition to F4, F5, and F6; Day 133: start of LCFA addition to F2, F3, F4, and F5

Biogas production patterns were markedly different for the sulfide-amended F4 and F5 digesters (Fig. 2c, d). In F4, a periodic rapid increase in biogas production was observed with the increasing oleate loading rate between days 133 and 208 (Fig. 1c). In contrast, stearate addition to F5 initially resulted in a decrease in biogas production of approximately 0.7 l d^−1^ compared with the control, but biogas production returned to a similar level as in F3 after the stearate loading rate increased (Fig. 1d). To further explore the microbial capacity for LCFA conversion, 0.67–4.0 g VS l^−1^ d^−1^ oleate and stearate (i.e., 50–400% of the PASS loading rate) were added over a short period between days 209 and 218. As a result, total biogas production increased to 9.0 l d^−1^ in F2 and 12 l d^−1^ in sulfide-amended F4, whereas it increased to 6.0 l d^−1^ in F3 and 6.5 l d^−1^ in sulfide-amended F5 (Fig. 2a, b). These results indicate that the degree of conversion of oleate to biogas at high loading rates was higher than that of stearate, and that sulfide addition further elevated daily biogas production from oleate during co-digestion with PASS. However, biogas production from LCFA did not exceed 30% of the theoretical daily biogas potential during the continuous addition phase (Fig. 2c, d), which indicates accumulation of LCFA in the digesters on day 218.

Between days 0 and 218, the VFA concentrations remained below the quantification limit of the analysis (< 0.6 mM) except in the sample collected from F4 on day 218, which contained 2.3 mM acetate and 1.0 mM propionate. Furthermore, pH remained constant at 7.4 ± 0.1 in all digesters. Total solids content of the digesters was 2.1–2.4% of total weight and VS content was 56–63% of TS. Gaseous hydrogen sulfide in F4, F5, and F6 increased from < 20 ppm to 440–510 ppm following addition of sulfide, while biogas from F1, F2, and F3 contained < 20 ppm hydrogen sulfide (Table 1). Addition of sulfide and LCFA did not result in any apparent changes in the methane content of the biogas, which ranged between 61 and 64% throughout the experiment (Table 1).

### Batch digestion of undegraded LCFA

**Fig. 3:**
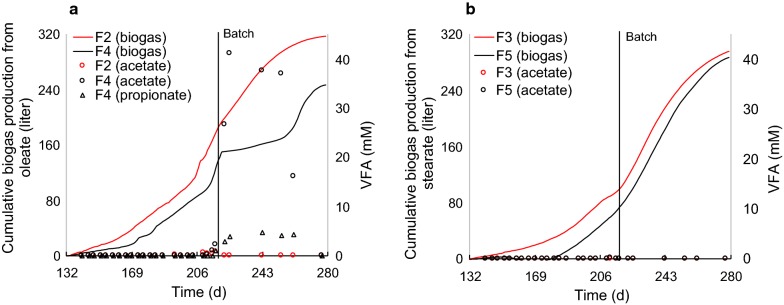
Cumulative biogas production after addition of long-chain fatty acids on day 133, together with volatile fatty acid (VFA) concentrations in the digesters. **a** Cumulative biogas productions from oleate, calculated by subtracting cumulative biogas production in digesters F1 and F6 from that in digesters F2 and F4, respectively. **b** Cumulative biogas productions from stearate, calculated by subtracting cumulative biogas production in digesters F1 and F6 from that in digesters F3 and F5, respectively. Theoretical biogas potential of total oleate and stearate was 482 and 479 l, calculated based on the Buswell equation [51]

Organic loading was stopped on day 219, to assess conversion of undegraded LCFA in the digesters during batch-mode digestion. Thereafter, biogas production from oleate in F2 steadily increased, yielding total cumulative biogas production of 318 l on day 280 (corresponding to 66% of the theoretical biogas potential of the total amount of oleate added; Fig. 3a). However, during the same period, biogas production in sulfide-amended F4 declined, with simultaneous accumulation of acetate to 40 mM and propionate to 4.6 mM (Fig. 3a). Towards the end of the experiment, acetate and propionate were depleted and biogas production resumed and reached 52% of the theoretical cumulative biogas potential of oleate (Fig. 3a). Accumulation of VFA was not observed for any of the other digesters. Biogas production in F3 and F5 also increased when the organic loading stopped and total cumulative biogas amount produced from stearate was 296 and 287 l, respectively (corresponding to 62% and 60% of the theoretical biogas potential of the total amount of stearate added; Fig. 3b).

### Bacterial community dynamics

Quality trim and chimera control of the raw data from sequencing of bacterial 16S rRNA genes yielded 6,459,998 reads for 78 triplicate samples, with 6920 to 89,353 sequences per sample (5th and 95th percentiles of 12,380 and 45,015 sequences per sample, respectively). The diversity and evenness indices (^1^D and ^1^E) for bacteria were 2–3 times higher in the inoculum compared with samples retrieved over the course of the experiment (Additional file 1: Table S1). In F1, which only received PASS, ^1^D and ^1^E remained relatively constant from day 72 onward. With increasing loading rate of oleate to F2 and F4, the diversity and evenness of bacteria increased, while with increasing loading rate of stearate to F3 and F5, the diversity indices changed only marginally (Additional file 1: Table S1). As a general trend, the ^1^D and ^1^E values were significantly lower (*p *< 0.05, *t* test) for the samples from the sulfide-amended digesters (F4–F6), particularly during continuous feeding (between days 76 and 218). The highest values of ^1^D and ^1^E indices were found in samples collected from F4 at the end of the experiment and the lowest values in F5 and F6 samples retrieved after day 219, i.e., during the batch-mode digestion (Additional file 1: Table S1).

**Fig. 4:**
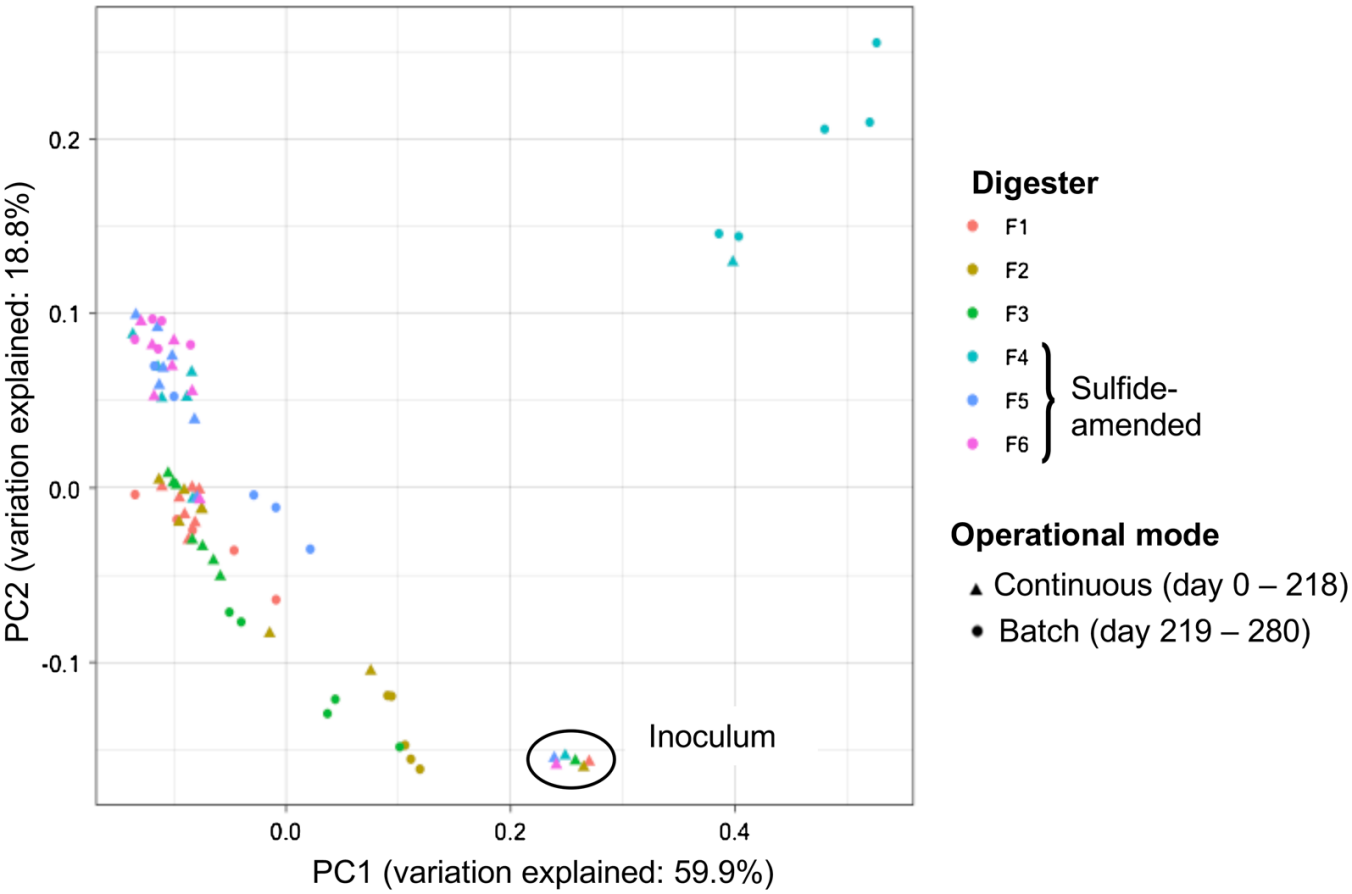
Phylogenetic distance of the bacterial communities, determined by weighted UniFrac principal coordinate (PC) analysis of the average ASV read counts from triplicate samples

Weighted UniFrac PCoA of the ASV read counts revealed that the inoculum samples grouped together and samples from sulfide-amended digesters formed a separate group from digesters that did not receive sulfide (Fig. 4). Bacterial community structure in LCFA-supplemented digesters (F2, F3, F4, and F5) gradually diverged following cessation of organic loading on day 219. Samples collected from F4 between days 208 and 280 showed the largest degree of dissimilarity, forming a separate cluster in the PCoA plot (Fig. 4). Accordingly, phylogenetic distance of the bacteria was primarily related to sulfide supplementation. A shift from continuous feeding to batch mode also contributed to alteration of the phylogenetic profiles, particularly in the LCFA-amended digesters.

**Fig. 5:**
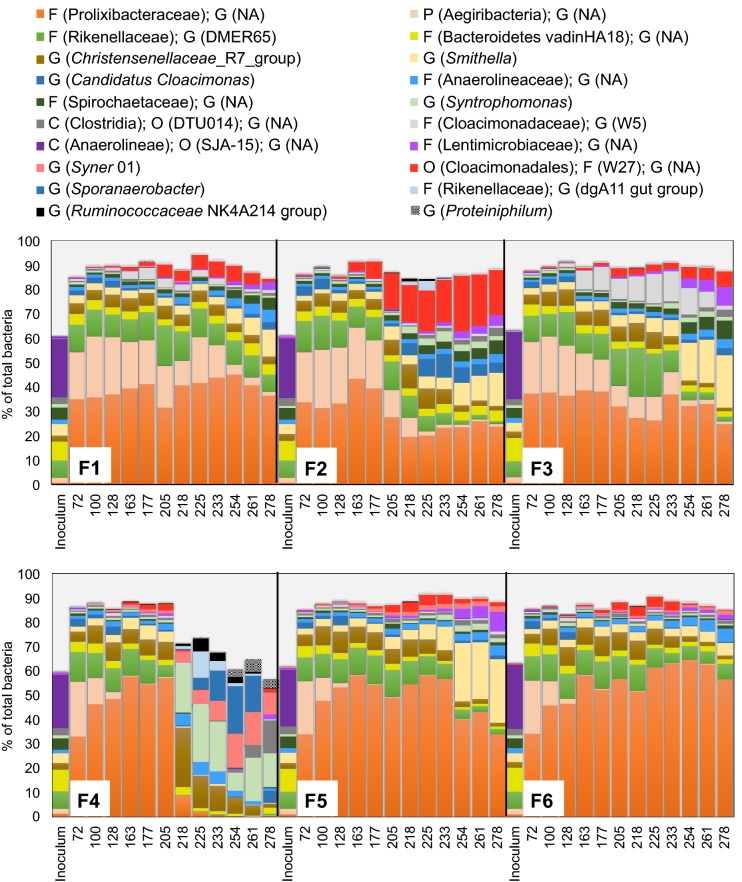
Relative abundances of bacterial 16S rRNA genes at genus (G) level based on the average ASV reads from triplicate samples, collected at different days from F1, F2, F3, F4, F5, and F6 digesters. Where genus name could not be assigned (NA) to the sequences, the closest classified taxonomic level is presented; phylum (P), class (C), order (O), family (F). Bacteria with relative abundance > 5% of total bacteria on at least one sampling occasion are depicted

Additional file 1: Figure S1 illustrates the dynamics of the bacterial community at phylum level, while Fig. 5 depicts the major taxonomic subgroups within the phyla, identified based on bacterial ASV sequence reads. The ASV sequences assigned to the phylum Chloroflexi, represented only by the order SJA-15 within the Anaerolineae class, dominated the bacterial community in the inoculum (25 ± 2.0% of total bacterial sequences). On day 72, the relative abundance of this phylum was < 2.0% of total bacteria, while members of the phyla Bacteroidetes (primarily the families Prolixibacteraceae, Bacteroidetes_vadinHA17, and Rikenellaceae) and Aegiribacteria prevailed in all digesters (52–55% and 19–22% of total bacteria, respectively; Fig. 5 and Additional file 1: Figure S1). Following sulfide addition on day 76, the relative abundance of Aegiribacteria decreased to < 1.0% of total bacteria in F4, F5, and F6 (Fig. 5). Bacteria present in relatively high abundances during start-up and after sulfide addition to the digesters belonged to the phyla Firmicutes, Cloacimonetes, Proteobacteria, and Spirochaetes, represented mainly by genera *Christensenellaceae* R7 group, *Candidatus Cloacimonas*, *Smithella*, and family Spirochaetaceae, respectively (Fig. 5).

During continuous addition of oleate to F2 from day 133 to 218, the relative abundance of the family W27 (order Cloacimonadales) and the genus *Candidatus Cloacimonas* (family Cloacimonadaceae), both belonging to the phylum Cloacimonetes, increased from < 1.0% to 15 ± 1.0% and from 1.6 ± 0.2 to 4.7 ± 0.2% of total bacteria, respectively (Fig. 5). There was also an increase in relative abundance of members of the phylum Firmicutes, i.e., *Christensenellaceae* R7 group (5.0 ± 0.5 to 9.7 ± 0.6% of total bacteria) and *Syntrophomonas* (< 1.0 to 2.2 ± 0.4% of total bacteria). Concomitantly, relative abundance of the phyla Bacteroidetes and Aegiribacteria declined from 51 ± 1.0 to 35 ± 2.0 and from 23 ± 2.0 to 7.9 ± 1.1% of total bacteria, respectively, upon oleate addition to F2 (Additional file 1: Figure S1). The relative abundance of *Smithella* (belonging to the phylum Proteobacteria) in oleate-amended F2 increased substantially, from 3.7 ± 0.4 to 14 ± 1.0% of total bacteria, between days 219 and 280, i.e., during the batch-mode digestion.

Dynamics of the bacterial community in sulfide-amended F4 showed a different pattern after oleate addition compared to F2. In F4, oleate supplementation and consequent VFA accumulation were associated with an increase in abundance of members of the phylum Firmicutes (Additional file 1: Figure S1), specifically the genera *Sporanaerobacter* (from < 1.0 to 20 ± 1.0% of total bacteria), *Christensenellaceae* R7 group (from 7.7 ± 0.9 to 25 ± 1.0% of total bacteria), and *Syntrophomonas* (from < 1.0 to 20 ± 2.0% of total bacteria). With the resumption of biogas production and depletion of acetate in F4 between days 250 and 280, the relative abundance of unidentified species of *Syntrophomonas* declined, whereas ASV sequences assigned to *Syntrophomonas wolfei* and *Syntrophomonas zehnderi* increased substantially (< 1.0 to 13 ± 0.0% and < 1.0 to 3.2 ± 0.1% of total bacteria, respectively; Additional file 1: Figure S2).

Stearate supplementation to F3 (no added sulfide) resulted in an increase in relative abundance of members of the phyla Cloacimonetes and Bacteroidetes, represented by the genera W5 (family Cloacimonadaceae; 1.3 ± 0.1 to 10 ± 1.0% of total bacteria) and DMER65 (family Rikenellaceae; 14 ± 3.0 to 20 ± 1.0% of total bacteria), respectively. The dynamics of the microbial community following stearate addition to sulfide-amended F5 were comparable to those in F6 (sulfide-amended control), but a minor increase in abundance of the family W27 (order Cloacimonadales; < 1.0 to 3.8 ± 0.3% of total bacteria) and the genus *Smithella* (2.4 ± 0.2 to 3.8 ± 0.4% of total bacteria) was observed. Analysis of indicator species in the samples based on the bacterial ASV read counts and frequency of occurrence identified five subgroups of bacteria (from a total of 196 taxa) that could be associated with different operating phases of the digesters (Additional file 1: Table S2). Among the abundant taxa, occurrences of the genera *Sporanaerobacter* and *Syntrophomonas* were significantly associated with periods of VFA accumulation during batch digestion of oleate in sulfide-amended F4 (days 218–275). Furthermore, *Candidatus Cloacimonas* and *Smithella*, together with the family W27 (order Cloacimonadales), were significantly associated with samples collected from F1, F3, and F5 towards the end of the experiment (days 254–278) and with samples collected from oleate-amended F2 throughout the batch phase (days 218–280; Additional file 1: Table S2).

### Archaeal community dynamics

**Fig. 6:**
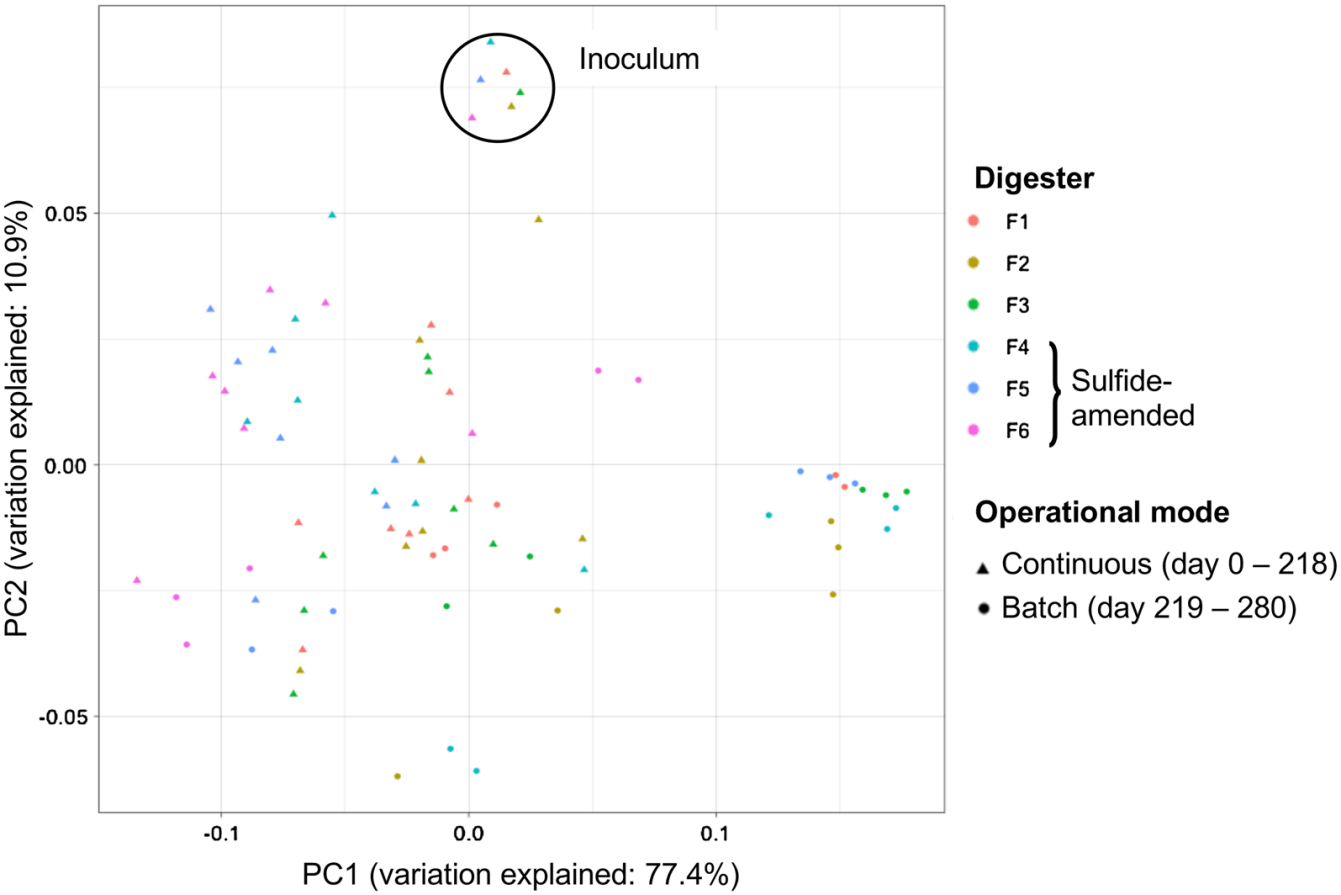
Phylogenetic distance of the archaeal communities, determined by weighted UniFrac principal coordinate (PC) analysis of archaeal ASV read counts

A total number of 1,303,939 sequences were acquired from 78 triplicate DNA samples after quality trim and chimera check of the archaeal sequence reads, with 2283 to 12,970 sequences per sample (5th and 95th percentiles of 2960 and 8644 sequences per sample, respectively). Similar to the bacterial community, the diversity and evenness of the archaea were substantially lower on day 72 compared with inoculum (Additional file 1: Table S3). The ^1^D and ^1^E values for archaea increased following addition of sulfide and were generally higher for the samples from sulfide-amended digesters (F4–F6) during continuous feeding. The archaeal diversities were comparable among the digesters during the batch phase. However, the archaea evenness showed an increasing trend towards the end of the experiment, except for F4 samples, in which it declined between days 261 and 278 (Additional file 1: Table S3). Weighted UniFrac PCoA of archaea phylogenetic distances showed separation of inoculum from the other samples (Fig. 6). Furthermore, samples from sulfide-amended digesters positioned more closely to each other in the UniFac PCoA plot than samples from digesters that did not receive sulfide. A higher degree of dissimilarity in archaeal ASV reads was also observed for the samples collected during batch mode compared with other samples (Fig. 6). In line with the results of the bacterial 16S rRNA genes sequencing, phylogenetic distance of the archaea was related to sulfide supplementation and to a shift from continuous feeding to batch-mode operation of the digesters.

**Fig. 7:**
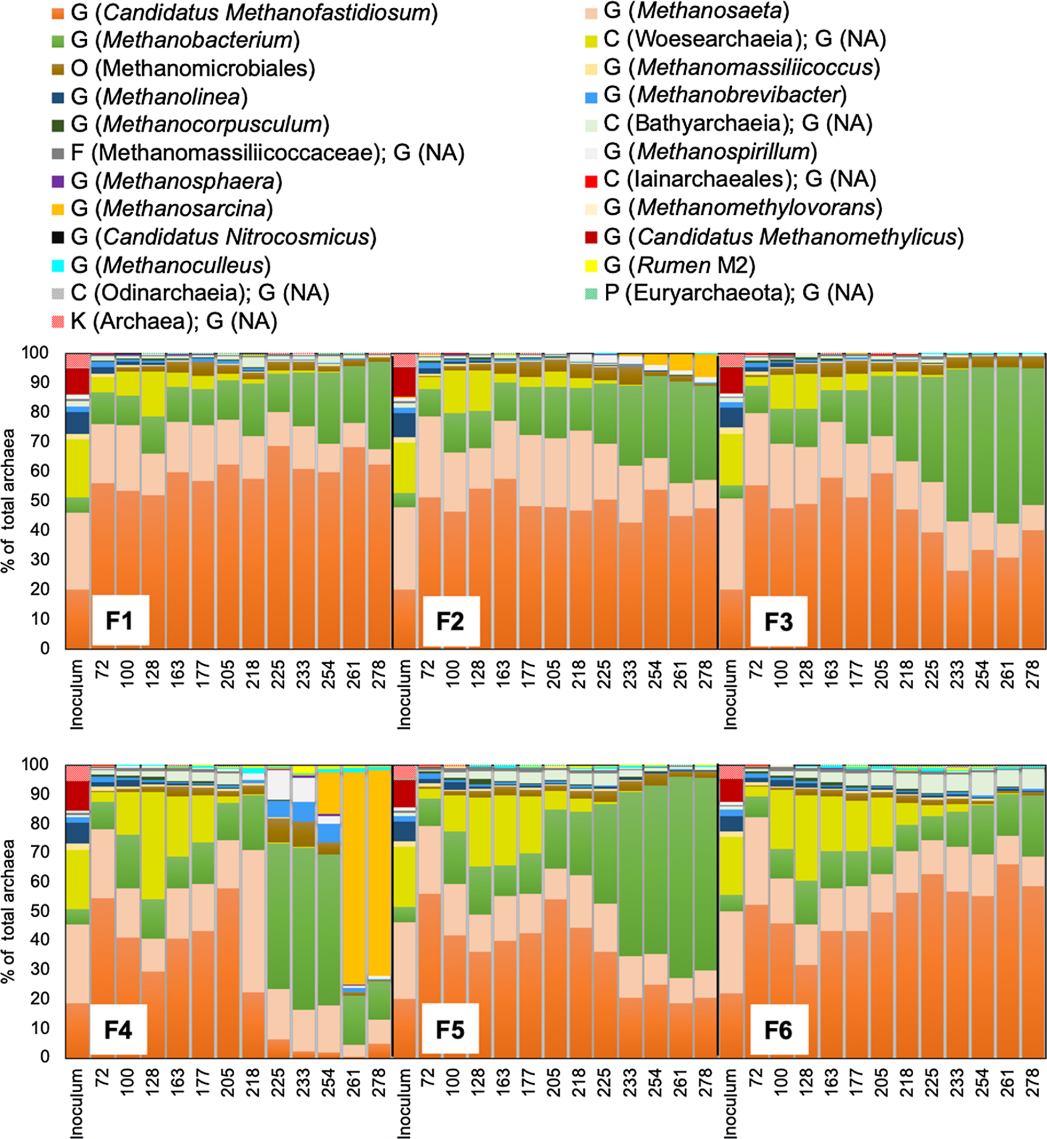
Relative abundances of archaeal 16S rRNA genes at genus (G) level based on the average ASV reads from triplicate samples collected on different days from digesters F1, F2, F3, F4, F5, and F6. Where genus name could not be assigned (NA) to the sequences, the closest classified taxonomic level is presented; kingdom (K), phylum (P), class (C), order (O), family (F)

Additional file 1: Figure S3 illustrates the dynamics of the archaeal community at phylum level, while Fig. 7 depicts the major taxonomic subgroups within the phyla, identified based on the archaeal ASV sequence reads. The archaeal community in the inoculum was dominated by the phyla Euryarchaeota (66 ± 2.0% of total archaea), Nanoarchaeaeota (19 ± 2.0% of total archaea), and Crenarchaeota (11 ± 1.0% of total archaea; Additional file 1: Figure S3). In the inoculum, the phylum Euryarchaeota mainly consisted of the genera *Methanosaeta* (class Methanomicrobia; 28 ± 2.0% of total archaea), *Candidatus Methanofastidiosum* (class Thermococci; 20 ± 1.0% of total archaea), *Methanolinea* (class Methanomicrobia; 6.8 ± 0.9% of total archaea), and *Methanobacterium* (class Methanobacteria; 5.2 ± 0.5% of total archaea). The phyla Nanoarchaeaeota and Crenarchaeota were represented by the class Woesearchaeia and the genus *Candidatus Methanomethylicus* (class Verstraetearchaeia), respectively. *Candidatus Methanofastidiosum* became dominant in all digesters, reaching relative abundance levels > 50% of total archaea on day 72 (Fig. 7). Upon addition of sulfide to F4, F5, and F6, the relative abundance of this genus temporarily declined (from approximately 50–30% of total archaea), but it returned to its former level by day 218 (Fig. 7). Moreover, *Candidatus Methanofastidiosum* abundance drastically declined, to < 1.0% of total archaea, in F4 following the overload of oleate on day 209, and instead *Methanosaeta* temporarily dominated the archaeal community (49 ± 2.0% of total archaea on day 218). Thereafter, a short-term rise in *Methanobacterium* abundance to 56 ± 2.0% of total archaea was observed in F4, in parallel with accumulation of VFA and the concomitant decline in daily biogas production. This was followed by increasing prevalence of *Methanosarcina* (to 73 ± 4.0% of total archaea) upon resumption of biogas production towards the end of the experiment (Fig. 7).

The relative abundance of *Methanobacterium* in the LCFA-amended digesters showed increasing trends throughout the experiment (Fig. 7), and went up more rapidly when the organic loading stopped on day 219. Representatives of the class Woesearchaeia were initially present in digesters (up to 37% of total archaea), but gradually declined in abundance towards the end of the experiment (Fig. 7). Analysis of indicator species revealed six archaeal groups, containing a total of 19 taxa, based on archaeal ASV read counts and frequency of occurrence (Additional file 1: Table S4). Among the abundant archaea, *Methanobacterium* and Methanomicrobiales were significantly associated with samples collected during the batch phase of the LCFA-amended digesters, while *Methanosarcina* was significantly associated with samples retrieved from F4 during depletion of VFA and resumption of biogas production (Additional file 1: Table S4).

## Discussion

Assessment of the operating performance of the different digesters showed that stearate and oleate conversion to biogas mainly occurred during the batch digestion phase (days 218–280). In line with our observations, enhanced LCFA degradation upon changing the operating mode from continuous feeding to batch has been reported previously [29]. Biogas production in the oleate- and sulfide-amended digester (F4) periodically and temporarily increased with stepwise increase of the oleate loading, which was followed by extensive acetate accumulation (Figs. 2, 3). Acetate and hydrogen are the major end-products of β-oxidation of even-numbered LCFA [30]. Accumulation of acetate to up to 40 mM in F4 when the PASS feeding was stopped suggests that the rate of oleate β-oxidation exceeded the rate of acetate conversion to biogas. This may have been due to faster β-oxidation of oleate to acetate and/or a lower conversion rate of acetate to biogas (e.g., caused by sulfide or LCFA inhibition of methanogens). Acetate accumulation did not occur in digester F2, which received a similar load of oleate but without added sulfide, or in F3 (stearate-amended), F5 (sulfide- and stearate-amended), or F6 (sulfide-amended control). Furthermore, unidentified species of LCFA-degrading *Syntrophomonas* increased in relative abundance in sulfide-amended F4, but not in the other digesters, directly after the oleate overload and the concomitant acetate accumulation (days 218, 225 and 223), which provide evidence for an enhanced oleate β-oxidation. It is, therefore, reasonable to suggest that sulfide addition to digester F4 had a stimulating effect on β-oxidation of oleate to acetate, while addition of both sulfide and oleate imposed a synergistic limiting effect on acetoclastic methanogenesis.

Basic local alignment search for unidentified members of *Syntrophomonas* gene sequences revealed 99.6% sequence similarity to *Syntrophomonas sapovorans* (strain OM), which is capable of β-oxidation of even-numbered saturated and unsaturated LCFA to acetate, propionate, and hydrogen [31]. Abundance of this species of *Syntrophomonas* increased in F4 together with members of *Methanobacterium*. Syntrophic association of species belonging to *Syntrophomonas* and *Methanobacterium* (i.e., *S. zehnderi* and *M. formicicum*) during anaerobic degradation of oleate has been observed previously [32], suggesting potential establishment of a syntrophic partnership between these microorganisms in F4. Towards the end of the experiment, the abundance of *Methanobacterium* and *S. sapovorans* declined with the depletion of acetate and resumption of biogas production in F4, whereas the abundance of *S. wolfei* and *S. zehnderi* increased (Additional file 1: Figure S2). These observations indicate potential involvement of physiologically diverse species of *Syntrophomonas* during β-oxidation of oleate in PASS digesters. Considering the substrate utilization patterns, the initial degradation of oleate in F4 was likely performed by *S. sapovorans*, which is capable of degrading C_4_–C_18_ organic acids [31]. In the later phase of F4 operation, oleate degradation was supported mainly by *S. wolfei*, which utilizes shorter acids (C_4_–C_8_) as the substrate [30]. In addition to *Syntrophomonas* species, *Sporoanaerobacter acetigenes* was also highly abundant in later phase of the F4 operation. This bacterium can utilize sugars and various amino acids via Strickland reaction, and uses elemental S as electron acceptor while producing acetate and hydrogen as the main products [33]. Thus, it seems unlikely that *S. acetigenes* was involved in the degradation of oleate, yet it might have contributed to formation of acetate and hydrogen in the sulfide- and oleate-amended F4.

Higher sulfide and oleate levels might have favored the prevalence of *Syntrophomonas* species (i.e., *S. wolfei*, *S. zehnderi*, and *S. sapovorans*) in F4. It has been suggested that sulfide contributes as a substrate to *O*-acetylserine(thiol)lyase activity, involved in biosynthesis of cysteine [34], which is in turn a precursor for biosynthesis of coenzyme A [35]. Although the direct mechanism of oleate and/or sulfide effects on metabolic functions of *Syntrophomonas* cannot be discerned, it maybe speculated that involvement of S-containing enzymes in β-oxidation pathway, e.g., enoyl-CoA hydratase and acyl-CoA dehydrogenases [36], during oleate metabolism by the *Syntrophomonas* species might have been benefited by the availability of sulfide in the digesters environment.

As the hydrogenotrophic *Methanobacterium* began to prevail in F4, the relative abundance of *Methanosaeta* declined to a level similar to that in the other digesters, following a temporary increase (Fig. 7). Thereafter, a rapid increase in the relative abundance of *Methanosarcina* along with the resumption of biogas production in F4 was observed, while *Methanosaeta* abundance decreased to approximately 5% of total archaea. It is well known that *Methanosarcina* growth is favored over *Methanosaeta* growth at high acetate concentrations, due to possessing a higher acetate threshold concentration [37], which may explain the pronounced increase in its relative abundance in F4 after the period of acetate accumulation (Fig. 7).

Following oleate and stearate addition to digesters F2 and F3, respectively, the relative abundance of Cloacimonetes increased. Among the members of this phylum, the family W27 (order Cloacimonadales) became abundant in F2, whereas sequences assigned to the genus W5 (family Cloacimonadaceae) increased in relative abundance in F3 (Fig. 5). Cloacimonetes is commonly prevalent in anaerobic environments, including anaerobic digesters [38]. Putative involvement of Cloacimonetes in lipid and oil degradation has been reported, based on enrichment of this phylum during acclimation of anaerobic cultures to lipid-rich wastes, with VFA (≤ C8 fatty acids) and hydrogen as the products [39, 40]. Furthermore, the genes involved in obligate syntrophic oxidation of propionate into acetate and carbon dioxide in the presence of hydrogen scavengers have been identified in members of phylum Cloacimonetes pointing at their syntrophic lifestyle [41]. Accordingly, the increase in relative abundance of Cloacimonetes in F2 and F3 following LCFA addition also suggests that these bacteria might be involved in syntrophic β-oxidation of LCFA, with different members taking part in degradation of saturated and unsaturated LCFA, i.e., genus W5 and family W27, respectively.

Interestingly, there was a distinct association of *Syntrophomonas* with anaerobic degradation of oleate, but not stearate. This shows that the members of this genus, which commonly prevails in lipid-fed anaerobic digesters [29], did not respond to the addition of saturated LCFA in our experiment. Instead, we observed increasing abundance of hydrogen-producing *Smithella*, together with higher prevalence of *Methanobacterium*, during conversion of stearate to biogas (digesters F3 and F5) in later stages of the experiment (Figs. 5, 7). As hydrogen is the product of the β-oxidation of stearate, an increase in the abundance of hydrogenotrophic *Methanobacterium* is likely related to the higher availability of hydrogen. An increase in relative abundance of *Smithella* may instead relates to the low hydrogen-sensitivity of this genus, as suggested during propionate oxidation in mixed anaerobic cultures [42, 43], and the consequent thermodynamic advantage over other hydrogen-sensitive organic acid oxidizers. Members of *Smithella* has also the ability to dismutate propionate to butyrate and acetate [44], which might also contribute to prevalence of this group during β-oxidation of the stearate under which the hydrogen-dependent propionate oxidation pathways have been thermodynamically limited at an elevated hydrogen partial pressure. Association of *Smithella* with alkane degradation has also been proposed [45, 46], yet the direct involvement of *Smithella* in LCFA degradation is unclear. *Smithella* belongs to the Syntrophaceae family, with phylogenetic proximity to the LCFA-degrading *Syntrophus* [45], which suggests potential for its involvement in β-oxidation of LCFA.

In addition, basic local alignment search for *Methanobacterium* identified different species in the digesters. During oleate degradation in F4 and an increase in the relative abundance of *Syntrophomonas*, the dominating species of *Methanobacterium* was most closely related to *M. beijingense* (98.0% sequence similarity). On the hand, members most closely related to *M. petrolearium* and *M. ferruginis* (98.8% sequence similarity) were likely dominant in LCFA-supplemented F2, F3, and F5, where *Smithella* prevailed in the batch phase (Figs. 5, 7). Members of *Methanobacterium* are able to use both format and hydrogen, yet there are differences between different species. For example, *M. beijingense* uses both format and hydrogen, while *M. petrolearium* and *M. ferruginis* are restricted to hydrogen utilization [47]. Accordingly, occurrences of different *Methanobacterium* species in the oleate- and sulfide-amended digester compared to the other digesters may suggest an establishment of different hydrogen and format levels.

Phylogenetic distances among the microbial community in the samples primarily related to the sulfide level (Figs. 4, 6). In particular, the initially dominant phylum Aegiribacteria disappeared from the microbial community in F4, F5, and F6 directly after addition of sulfide (Fig. 5). Aegiribacteria has recently been discovered in a lake sediment with unconventionally high sulfate and sulfide levels, purportedly as fermentative bacteria belonging to the superphylum Fibrobacteres–Chlorobi–Bacteroidetes [48]. However, our results indicate sensitivity of Aegiribacteria to elevated sulfide level in PASS digesters, since the relative sequence abundance of this phylum declined in digesters F4–F6 directly after addition of sulfide.

Based on our observations, the effect of sulfide on the microbial community was more pronounced for oleate than for stearate loading. The presence of sulfide at high levels promotes the availability and concentration of various S species, including hydrogen sulfide ions and polysulfides [14]. The C=C and C=O double bonds in the carbon skeleton of organic matter are particularly susceptible to reaction with inorganic sulfide species to form organic S compounds (i.e., sulfurization) [49]. Furthermore, organic and inorganic sulfide ions (HS^−^) can be converted into radicals in the presence of transition metals, such as Fe that is often abundant in PASS-fed digesters [12], which may act as a catalyst for *cis*–*trans* isomerization of unsaturated LCFA [50]. Unsaturated fatty acids in cis isomeric state have lower flexibility than their saturated or *trans* counterparts, which restrain the area of the monolayers in LCFA, with implications for their preferability as a substrate for bacteria [50]. Thus, the pronounced effect of sulfide on oleate conversion in F4 may partly relate to abiotic alteration of the unsaturated LCFA structure (e.g., via sulfurization and/or stereomutation), along with physiological effects of the elevated sulfide level on microorganisms in the digesters. To our knowledge, information on the occurrence and extend of the abiotic interactions of sulfide with microbial organic matter degradation in the anaerobic digester environments is scarce. Accordingly, further research is needed to distinguish the abiotic effects of biogenic sulfide on the chemical properties and degradability of LCFA as well as their kinetic and thermodynamic feasibility in the anaerobic digesters.

## Conclusions

This study shows that LCFA chain saturation and sulfide level are selective drivers for establishment of LCFA-degrading bacteria and methanogenic communities in municipal sludge digesters. In this regard, microbial community structure and function during co-digestion of lipid wastes with PASS are influenced by composition of the LCFA in terms of fatty acid chain saturation. Furthermore, conversion of unsaturated LCFA (oleate) to mainly acetate was promoted at elevated sulfide level, most likely due to prevalence of LCFA-degrading *Syntrophomonas*. This observation suggests potential for application of S-rich substrates together with waste lipids to improve the capacity of the microbial community in PASS digesters for β-oxidation of unsaturated LCFA. However, supplementation of sulfide and high loads of oleate imposed a synergistic limiting effect on acetoclastic methanogenesis, pointing at the importance of optimizing lipid load relative to sulfide levels in anaerobic digesters to avoid such effects. Finally, higher abundances of hydrogenotrophic methanogens were associated with improved conversion of LCFA to biogas in the digesters, highlighting the need for establishment of hydrogen-utilizing methanogens to achieve efficient LCFA conversion in PASS digesters.

## Supplementary information

**Additional file 1.** (Additional results): **Table S1.** First-order Hill’s diversity and evenness of bacterial communities. **Table S2.** Indicator bacteria in the samples. **Table S3.** First-order Hill’s diversity and evenness of archaeal communities. **Table S4.** Indicator archaea in the samples. **Figure S1.** Relative abundances of 16S rRNA genes of bacterial phyla. **Figure S2.** Relative abundances of 16S rRNA genes assigned to species in the genus *Syntrophomonas*. **Figure S3.** Relative abundances of 16S rRNA genes of archaeal phyla.

## Data Availability

The raw sequencing data are available at the National Center for Biotechnology Information database, under identification number SRP188169.

## Abbreviations

AD: anaerobic digestion
ASV: amplicon sequence variants
LCFA: long-chain fatty acids
OLR: organic loading rate
PASS: primary and activated sewage sludge
PCoA: principal coordinate analysis
PCR: polymerase chain reaction
TS: total solid
VFA: volatile fatty acids
VS: volatile solid
WWTP: wastewater treatment plant
