# Supplementary material for: Sulfide level in municipal sludge digesters affects microbial community response to long-chain fatty acid loads
Source: Biotechnol Biofuels. 2019 Nov 2;12:259. doi: 10.1186/s13068-019-1598-1 (PMC6825336; doi:10.1186/s13068-019-1598-1)
Supplement: Supplementary file 1 — Additional file 1. (Additional results): Table S1. First-order Hill’s diversity and evenness of bacterial communities. Table S2. Indicator bacteria in the samples. Table S3. First-order Hill’s diversity and evenness of archaeal communities. Table S4. Indicator archaea in the samples. Figure S1. Relative abundances of 16S rRNA genes of bacterial phyla. Figure S2. Relative abundances of 16S rRNA genes assigned to species in the genus Syntrophomonas. Figure S3. Relative abundances of 16S rRNA genes of archaeal phyla. [file 13068_2019_1598_MOESM1_ESM.docx]

**Additional file 1 (supporting results)**

**Sulfide level in municipal sludge digesters affects microbial community response to long-chain fatty acid loads**

Sepehr Shakeri Yekta^1,*^, Tong Liu^2^, Mette Axelsson Bjerg^1^, Luka Šafarič^1^, Anna Karlsson^3^, Annika Björn^1^, Anna Schnürer^1,2^

*^1^Department of Thematic Studies-Environmental Change and Biogas Research Center, Linköping University, 581 83 Linköping, Sweden*

*^2^Department of Molecular Sciences, Swedish University of Agricultural Sciences, Uppsala BioCenter, 75007 Uppsala, Sweden*

*^3^Scandinavian Biogas Fuels AB, Stockholm, Sweden.*

**Correspondence:* [*sepehr.shakeri.yekta@liu.se*](mailto:sepehr.shakeri.yekta@liu.se)*; Tel.: +46-13-282-294*

**Table S1 First-order Hill’s diversity (^1^D) and evenness (^1^E) of the bacterial community in samples collected in different operational phases of digesters F1-F6 (n=3, mean ± standard deviation)**

|  |  |  | **Bacteria** |  |  |  |
| --- | --- | --- | --- | --- | --- | --- |
| **Day** | **F1-^1^D** | **F2-^1^D** | **F3-^1^D** | **F4-^1^D** | **F5-^1^D** | **F6-^1^D** |
| 0 | 77±14 | 70±3.4 | 65±2.6 | 78±5.4 | 72±15 | 66±7.0 |
| 72 | 25±4.2 | 24±1.6 | 24±2.8 | 26±4.0 | 26±2.6 | 27±4.5 |
| 100 | 23±2.3 | 25±0.5 | 24±2.1 | 21±2.5 | 21±4.2 | 23±4.0 |
| 128 | 23±1.4 | 26±0.5 | 20±2.3 | 25±9.5 | 18±2.8 | 27±6.8 |
| 163 | 24±0.5 | 20±0.1 | 26±4.3 | 15±1.2 | 15±2.3 | 16±1.2 |
| 177 | 23±0.6 | 21±1.7 | 22±0.4 | 17±1.9 | 16±1.1 | 20±1.8 |
| 205 | 27±5.1 | 34±4.8 | 34±5.1 | 16±1.4 | 23±1.5 | 12±1.5 |
| 218 | 28±0.9 | 47±5.2 | 32±1.9 | 50±3.3 | 17±3.2 | 19±3.6 |
| 225 | 18±0.6 | 47±3.8 | 30±1.2 | 43±1.6 | 13±2.6 | 8±0.2 |
| 233 | 21±3.4 | 46±8.2 | 25±4.4 | 45±0.3 | 14±2.6 | 12±1.4 |
| 254 | 24±3.2 | 35±1.5 | 29±1.4 | 41±1.6 | 19±3.3 | 11±1.7 |
| 261 | 25±0.5 | 35±1.4 | 30±3.8 | 42±0.9 | 16±0.9 | 11±1.1 |
| 278 | 28±0.9 | 33±10 | 34±3.6 | 63±3.2 | 20±1.6 | 12±0.3 |
| **Day** | **F1-^1^E** | **F2-^1^E** | **F3-^1^E** | **F4-^1^E** | **F5-1E** | **F6-^1^E** |
| 0 | 0.37±0.06 | 0.37±0.02 | 0.37±0.01 | 0.40±0.01 | 0.41±0.01 | 0.37±0.03 |
| 72 | 0.18±0.01 | 0.17±0.01 | 0.15±0.01 | 0.15±0.01 | 0.15±0.01 | 0.15±0.00 |
| 100 | 0.15±0.02 | 0.17±0.02 | 0.18±0.02 | 0.14±0.03 | 0.14±0.01 | 0.13±0.01 |
| 128 | 0.14±0.01 | 0.17±0.00 | 0.18±0.02 | 0.13±0.01 | 0.12±0.00 | 0.14±0.01 |
| 163 | 0.13±0.01 | 0.11±0.01 | 0.14±0.00 | 0.09±0.01 | 0.09±0.00 | 0.09±0.01 |
| 177 | 0.13±0.01 | 0.16±0.01 | 0.17±0.01 | 0.11±0.01 | 0.11±0.02 | 0.11±0.01 |
| 205 | 0.18±0.01 | 0.20±0.02 | 0.15±0.01 | 0.07±0.01 | 0.10±0.01 | 0.11±0.00 |
| 218 | 0.13±0.01 | 0.27±0.01 | 0.17±0.01 | 0.28±0.00 | 0.09±0.01 | 0.11±0.01 |
| 225 | 0.14±0.01 | 0.24±0.01 | 0.18±0.03 | 0.29±0.02 | 0.08±0.00 | 0.08±0.00 |
| 233 | 0.13±0.00 | 0.23±0.02 | 0.17±0.01 | 0.33±0.03 | 0.08±0.01 | 0.07±0.00 |
| 254 | 0.14±0.03 | 0.25±0.02 | 0.22±0.02 | 0.29±0.02 | 0.12±0.02 | 0.06±0.01 |
| 261 | 0.21±0.02 | 0.23±0.04 | 0.20±0.02 | 0.31±0.02 | 0.13±0.01 | 0.08±0.00 |
| 278 | 0.24±0.03 | 0.30±0.02 | 0.25±0.01 | 0.47±0.02 | 0.16±0.01 | 0.12±0.01 |

**Table S2 Indicator bacteria^a)^ in five clusters of samples, distinguished based on relative ASV read counts and frequency of occurrence**

| Cluster 1 | Cluster 2 | Cluster 3 | Cluster 4 | Cluster 5 |
| --- | --- | --- | --- | --- |
| Taxonomic level | **Taxonomic level** | **Taxonomic level** | **Taxonomic level** | **Taxonomic level** |
| r value | **r value** | **r value** | **r value** | **r value** |
| Aegiribacteria | *Saccharofermentans* | LNR_A2.18 | *Sporanaerobacter* | W27 |
| P | G | G | G | F |
| 0.80 | 0.42 | 0.63 | 1.0 | 0.64 |
| Family XIII | Spirochaetaceae | *Oligosphaera* | *Lactobacillus* | Lentimicrobiaceae |
| F | F | G | G | F |
| 0.64 | 0.39 | 0.57 | 0.93 | 0.61 |
| W5 | *Syntrophorhabdus* | *Ruminiclostridium* | *Syntrophomonas* | *Candidatus Cloacimonas* |
| G | G | G | G | G |
| 0.58 | 0.36 | 0.54 | 0.93 | 0.51 |
| Gracilibacter | Syntrophaceae | Marinimicrobia SAR406 clade | *Ruminococcaceae* NK4A214 group | Sphingobacteriales |
| F | F | P | G | O |
| 0.52 | 0.34 | 0.52 | 0.86 | 0.48 |
| M2PB4 65 termite group | Gracilibacter | Prolixibacteraceae | dgA.11 gut group | *Smithella* |
| F | F | F | G | G |
| 0.51 | 0.32 | 0.52 | 0.85 | 0.48 |
| *Sedimentibacter* | Armatimonadetes | p-251-o5 | *Proteiniphilum* | SBR1031 |
| G | P | F | G | O |
| 0.47 | 0.29 | 0.51 | 0.83 | 0.43 |
| *Peptoclostridium* | *Candidatus Caldatribacterium* | GZKB124 | *Fastidiosipila* | Chloroflexi |
| G | G | F | G | P |
| 0.41 | 0.29 | 0.51 | 0.82 | 0.42 |
| Prolixibacteraceae | *Leptolinea* | *Anaerosporobacter* | *Tetrasphaera* | ADurb.Bin063.1 |
| F | G | G | G | G |
| 0.36 | 0.26 | 0.46 | 0.82 | 0.40 |
| DMER64 | WCHB1.41 | Z20 | *Syner* 01 | *Bellilinea* |
| G | O | G | G | G |
| 0.35 | 0.23 | 0.44 | 0.8 | 0.35 |
| *Saccharofermentans* | p-251-o5 | GWE2-42-42 | *Herbinix* | Spirochaetaceae |
| G | F | G | G | F |
| 0.33 | 0.21 | 0.41 | 0.78 | 0.35 |

a) The 10 bacteria with the highest degree of significant association to each cluster are presented. When genus (G) name could not be assigned to the sequences, the closest classified taxonomic level is listed: Phylum (P), Order (O), Family (F). The indicator values, r, for the assigned taxa represent the degree of association to each cluster (max = 1). Samples associated to each cluster, digester name (sampling day):

- Cluster 1: F1 (72-233), F2 (72-205), F3 (72-233), F4 (72-100), F5 (72-100), F6 (72-100)
- Cluster 2: F1-F6 (inoculum)
- Cluster 3: F4 (128-205), F5 (128-233), F6 (128-278).
- Cluster 4: F4 (218-275)
- Cluster 5: F1 (254-278), F2 (218-278), F3 (254-278), F5 (254-278).

**Table S3 First-order Hill’s diversity (^1^D) and evenness (^1^E) of the archaeal community in samples collected at different operational phases of digesters F1-F6 (n=3, mean ± standard deviation)**

|  |  |  | **Archaea** |  |  |  |
| --- | --- | --- | --- | --- | --- | --- |
| **Day** | **F1-^1^D** | **F2-^1^D** | **F3-^1^D** | **F4-^1^D** | **F5-^1^D** | **F6-^1^D** |
| 0 | 7.7±0.4 | 7.6±0.2 | 7.2±0.4 | 7.4±0.4 | 7.3±0.1 | 7.2±0.1 |
| 72 | 4.0±0.2 | 4.1±0.1 | 3.9±0.1 | 4.1±0.1 | 3.9±0.1 | 3.8±0.1 |
| 100 | 4.0±0.2 | 4.5±0.3 | 4.5±0.1 | 5.2±0.2 | 5.3±0.3 | 4.8±0.3 |
| 128 | 4.3±0.3 | 4.1±0.2 | 4.3±0.1 | 5.2±0.2 | 5.6±0.1 | 5.6±0.1 |
| 163 | 3.6±0.1 | 3.6±0.1 | 3.7±0.3 | 5.3±0.1 | 5.2±0.0 | 5.3±0.1 |
| 177 | 3.8±0.3 | 4.0±0.0 | 4.0±0.1 | 5.3±0.2 | 5.2±0.2 | 5.4±0.2 |
| 205 | 3.4±0.1 | 4.0±0.2 | 3.4±0.0 | 4.1±0.1 | 4.2±0.2 | 4.9±0.3 |
| 218 | 3.8±0.1 | 4.1±0.1 | 3.8±0.1 | 4.1±0.1 | 5.0±0.1 | 4.6±0.2 |
| 225 | 2.9±0.2 | 4.0±0.2 | 3.9±0.2 | 4.8±0.3 | 4.7±0.1 | 4.0±0.2 |
| 233 | 3.3±0.1 | 4.1±0.0 | 3.3±0.1 | 4.5±0.5 | 3.7±0.1 | 4.4±0.1 |
| 254 | 3.2±0.1 | 3.4±0.1 | 3.2±0.0 | 4.6±0.1 | 3.2±0.2 | 4.1±0.3 |
| 261 | 2.6±0.1 | 3.7±0.0 | 3.1±0.1 | 2.6±0.2 | 2.6±0.3 | 3.2±0.1 |
| 278 | 2.6±0.2 | 3.6±0.2 | 3.1±0.1 | 2.8±0.2 | 2.7±0.0 | 3.5±0.2 |
| **Day** | **F1-^1^E** | **F2-^1^E** | **F3-^1^E** | **F4-^1^E** | **F5-1E** | **F6-^1^E** |
| 0 | 0.49±0.08 | 0.51±0.03 | 0.49±0.05 | 0.55±0.08 | 0.53±0.04 | 0.50±0.05 |
| 72 | 0.31±0.04 | 0.29±0.03 | 0.25±0.00 | 0.26±0.02 | 0.25±0.02 | 0.26±0.01 |
| 100 | 0.28±0.00 | 0.29±0.03 | 0.31±0.02 | 0.39±0.00 | 0.39±0.05 | 0.35±0.01 |
| 128 | 0.29±0.02 | 0.33±0.05 | 0.35±0.02 | 0.36±0.03 | 0.41±0.01 | 0.41±0.01 |
| 163 | 0.28±0.02 | 0.26±0.04 | 0.29±0.02 | 0.36±0.01 | 0.37±0.06 | 0.34±0.04 |
| 177 | 0.31±0.02 | 0.35±0.02 | 0.39±0.03 | 0.36±0.02 | 0.36±0.00 | 0.38±0.04 |
| 205 | 0.26±0.03 | 0.40±0.02 | 0.23±0.02 | 0.24±0.02 | 0.26±0.01 | 0.31±0.02 |
| 218 | 0.24±0.01 | 0.38±0.04 | 0.32±0.04 | 0.31±0.03 | 0.33±0.01 | 0.29±0.02 |
| 225 | 0.26±0.01 | 0.33±0.02 | 0.34±0.02 | 0.39±0.07 | 0.34±0.02 | 0.25±0.01 |
| 233 | 0.28±0.01 | 0.40±0.06 | 0.36±0.09 | 0.34±0.04 | 0.26±0.02 | 0.28±0.03 |
| 254 | 0.24±0.01 | 0.39±0.08 | 0.34±0.04 | 0.44±0.03 | 0.26±0.03 | 0.26±0.00 |
| 261 | 0.28±0.03 | 0.37±0.04 | 0.40±0.09 | 0.25±0.05 | 0.27±0.05 | 0.25±0.02 |
| 278 | 0.31±0.02 | 0.44±0.03 | 0.39±0.09 | 0.26±0.01 | 0.34±0.04 | 0.29±0.03 |

**Table S4 Indicator archaea in six clusters of samples, distinguished based on relative ASV read counts and frequency of occurrence**

| ^a)^Cluster number | 1 | 2 | 3 | 4 | 5 | 6 |
| --- | --- | --- | --- | --- | --- | --- |
|  | r | | | | | |
| *Candidatus Methanofastidiosum* | 0.30 | 0.24 | 0.20 | 0.10 |  | 0.15 |
| *Candidatus Methanomethylicus* |  |  |  | 1.0 |  |  |
| *Candidatus Nitrocosmicus* |  |  |  | 0.09 |  |  |
| *Methanobacterium* | 0.15 | 0.10 | 0.10 |  | 0.20 | 0.41 |
| *Methanobrevibacter* | 0.08 | 0.12 | 0.09 | 0.26 | 0.35 | 0.05 |
| *Methanocorpusculum* | 0.15 | 0.29 | 0.45 |  |  |  |
| *Methanoculleus* | 0.07 |  | 0.08 |  | 0.57 | 0.06 |
| *Methanolinea* |  |  | 0.09 | 0.79 |  |  |
| *Methanomassiliicoccus* | 0.09 | 0.16 | 0.14 | 0.52 |  |  |
| *Methanomethylovorans* | 0.15 | 0.06 | 0.12 |  |  |  |
| *Methanosaeta* | 0.13 | 0.22 | 0.15 | 0.28 | 0.09 | 0.13 |
| *Methanosarcina* |  |  |  |  | 0.97 |  |
| *Methanosphaera* |  | 0.14 |  |  | 0.25 |  |
| *Methanospirillum* |  | 0.07 |  | 0.27 | 0.28 | 0.24 |
| RumEn M2 | 0.07 |  |  |  | 0.62 | 0.05 |
| Methanomassiliicoccaceae | 0.20 | 0.11 | 0.37 | 0.21 |  |  |
| Iainarchaeales | 0.06 | 0.10 | 0.05 |  |  |  |
| Methanomicrobiales | 0.18 | 0.19 | 0.17 |  | 0.11 | 0.29 |
| Bathyarchaeia | 0.36 | 0.07 | 0.26 | 0.17 |  | 0.07 |
| Odinarchaeia |  |  |  | 0.89 |  |  |
| Woesearchaeia | 0.05 | 0.08 | 0.43 | 0.43 |  |  |

a) The indicator values, r, for the assigned taxa represent the degree of association to each cluster (max = 1). Samples associated to each cluster, digester name (sampling day):

- Cluster 1: F1 (205-278), F3 (205-218), F4 (205), F5 (205-218), F6 (218), F6 (225-278).
- Cluster 2: F1 (72-100, 163-177), F2 (72, 163-233), F3 (72-177), F4 (72, 218), F5 (72), F6 (72).
- Cluster 3: F1 (128), F2 (100-128), F4 (100 -177), F5 (100-177), F6 (100-205).
- Cluster 4: F1-F6 (inoculum)
- Cluster 5: F4 (254-278).
- Cluster 6: F2 (233-278), F3 (225-278), F4 (225), F4 (233), F5 (225-278).


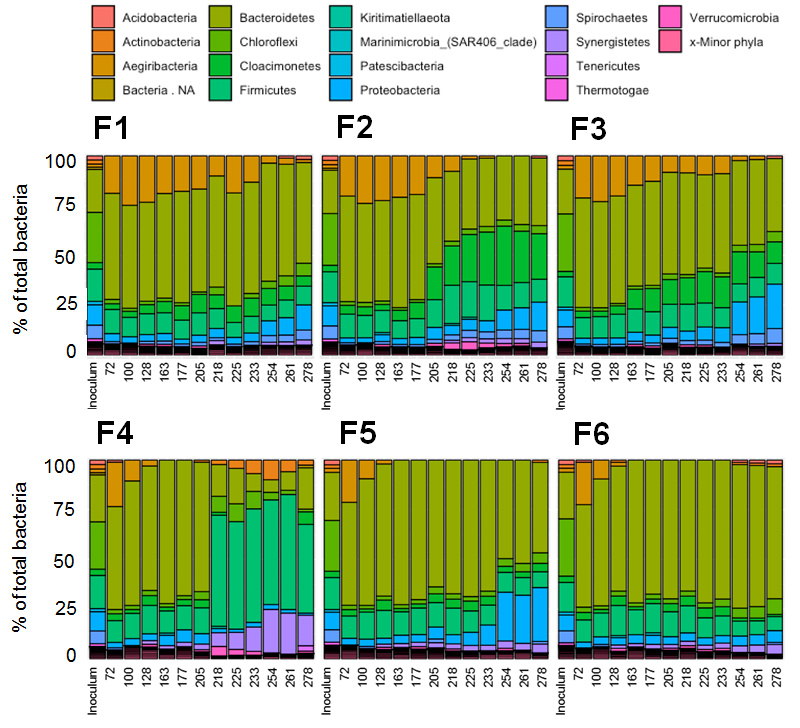


**Fig. S1** Relative abundances of 16S rRNA genes of bacterial phyla based on the average ASV reads from triplicate samples collected at different days from digesters F1, F2, F3, F4, F5, and F6 digesters. NA stands for not assigned.

**Fig. S2** Relative abundances of 16S rRNA genes assigned to unidentified species of the genus *Syntrophomonas* (NA) and the species *S. wolfei* and *S. zehnderi* based on the average ASV reads from triplicate samples collected at different days from digester F4. Basic local alignment search for the unidentified members of *Syntrophomonas* gene sequences revealed 99.6% sequence similarity to *Syntrophomonas sapovorans* (strain OM).


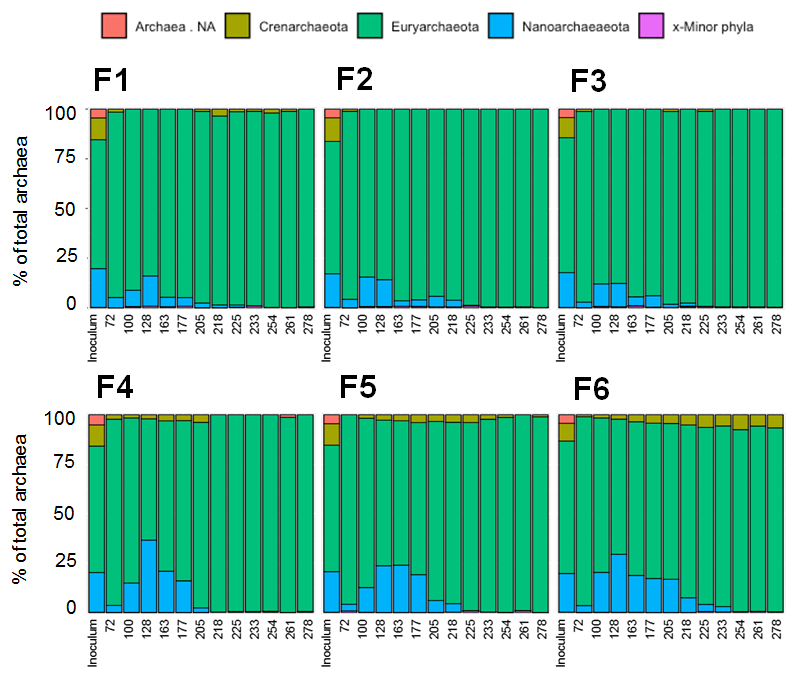


**Fig. S3** Relative abundances of 16S rRNA genes of archaeal phyla based on the average ASV reads from triplicate samples collected at different days from digesters F1, F2, F3, F4, F5, and F6 digesters. NA stands for not assigned.
